# 

**Published:** 1891-09

**Authors:** 


Bristol Medico-Chirurgical Journal.

SEPTEMBER, 1891.

CONTENTS.

ORIGINAL PAPERS: *Page*

Chloroform *versus* Ether. By W. H. HARSANT, F.R.C.S. Eng.... ... 145

Cases of Septic Thrombosis of the Lateral Sinus. By CHARLES F. PICKERING, F.R.C.S. Eng. ... ... ... ... ... ... ... ... ... ... 155

Case of Tumour of the Pons. By P. WATSON WILLIAMS, M.B. Lond. 163

Buxton: Its Baths and Climate. By ALFRED J. H. CRESPI, M.R.C.S. Eng., M.R.C.P.I. ... ... ... ... ... ... ... ... ... ... ... ... ... ... 167

PROGRESS OF THE MEDICAL SCIENCES ... ... ... ... ... ... ... ... 184

REVIEWS OF BOOKS ... ... ... ... ... ... ... ... ... ... ... ... ... ... 204

NOTES ON PREPARATIONS FOR THE SICK ... ... ... ... ... ... ... ... 213

THE LIBRARY OF THE BRISTOL MEDICO-CHIRURGICAL SOCIETY ... ... ... 215

SCRAPS ... ... ... ... ... ... ... ... ... ... ... ... ... ... ... ... 238

